# Vitamin D Levels in Women with Polycystic Ovary Syndrome: A Population-Based Study

**DOI:** 10.3390/nu11112831

**Published:** 2019-11-19

**Authors:** Johanna Lumme, Sylvain Sebert, Paula Pesonen, Terhi Piltonen, Marjo-Riitta Järvelin, Karl-Heinz Herzig, Juha Auvinen, Marja Ojaniemi, Maarit Niinimäki

**Affiliations:** 1 PEDEGO Research Unit (Research Unit for Pediatrics, Dermatology, Clinical Genetics, Obstetrics and Gynecology), University of Oulu, 90014 Oulu, Finland; terhi.piltonen@oulu.fi (T.P.); marja.ojaniemi@oulu.fi (M.O.); maarit.niinimaki@oulu.fi (M.N.); 2 Medical Research Center Oulu (MRC Oulu), University of Oulu, 90014 Oulu, Finland; karl-heinz.herzig@oulu.fi; 3 Department of Obstetrics and Gynecology, Oulu University Hospital, 90220 Oulu, Finland; 4 Department of Pediatrics and Adolescence, Oulu University Hospital, 90220 Oulu, Finland; 5 Center for Life-Course Health Research, Faculty of Medicine, University of Oulu, 90014 Oulu, Finland; sylvain.sebert@oulu.fi (S.S.); m.jarvelin@imperial.ac.uk (M.-R.J.); juha.auvinen@oulu.fi (J.A.); 6 Infrastructure for Population Studies, Faculty of Medicine, University of Oulu, 90014 Oulu, Finland; paula.pesonen@oulu.fi; 7 Biocenter Oulu, University of Oulu, 90014 Oulu, Finland; 8 Department of Epidemiology and Biostatistics, MRC Centre for Environment and Health, School of Public Health, Imperial College, London W2 1PG, UK; 9 Department of Life Sciences, College of Health and Life Sciences, Brunel University London, Kingston Lane, Uxbridge, Middlesex UB8 3PH, UK; 10 Institute of Biomedicine, University of Oulu, 90014 Oulu, Finland; 11 Department of Gastroenterology and Metabolism, Poznan University of Medical Sciences, 61-701 Poznan, Poland; 12 Oulunkaari Health Center, 91100 Ii, Finland

**Keywords:** vitamin D, 25(OH)D, polycystic ovary syndrome, population-based study, community setting, body mass index

## Abstract

Background: Conflicting evidence supports a role for vitamin D in women with reproductive disorders such as polycystic ovary syndrome (PCOS) but studies on large, unselected populations have been lacking. Methods: We conducted a general population-based study from the prospective Northern Finland Birth Cohort 1966 (NFBC1966). Serum 25-hydroksyvitamin D (25(OH)D) levels were evaluated in women with self-reported PCOS (*n* = 280) versus non-symptomatic controls (*n* = 1573) at the age of 31 with wide range of endocrine and metabolic confounders. Results: The levels of 25(OH)D were similar among women with and without self-reported PCOS (50.35 vs. 48.30 nmol/L, *p* = 0.051). Women with self-reported PCOS presented with a higher body mass index (BMI), increased insulin resistance, and low-grade inflammation and testosterone levels compared to controls. The adjusted linear regression model showed a positive association between total 25(OH)D levels in self-reported PCOS (β = 2.46, 95% confidence interval (CI) 0.84 to 4.08, *p* = 0.003). The result remained after adjustment for BMI, testosterone, homeostatic model assessment of insulin resistance (HOMA-IR), and high-sensitivity C-reactive protein (hs-CRP) levels. Conclusion: In this population-based setting, PCOS was associated with higher vitamin D levels when adjusting for confounding factors, without distinct beneficial effects on metabolic derangements.

## 1. Introduction

Polycystic ovary syndrome (PCOS) is the most common endocrine disorder in women of reproductive age [1]. It is characterized by polycystic ovaries, oligo-amenorrhea, and hyperandrogenism [1,2]. PCOS is commonly related to features of metabolic syndrome, including obesity, impaired glucose metabolism, and insulin resistance (IR) [3] and is one of the main causes of anovulatory infertility [4]. Current knowledge suggests that vitamin D may have a role in improving reproductive and metabolic health in affected women [5,6,7,8,9]. Indeed, the nuclear receptor for vitamin D (VDR) is expressed in several tissues, including the ovaries, suggesting a regulatory function. However, the precise action of vitamin D in PCOS is unknown [5,10,11], and studies addressing vitamin D levels have been inconclusive: Lower, similar, and higher concentrations have been reported in women with PCOS [6,9,12]. Given the high rate of obesity among women with PCOS and the fact that, in the general population, a low concentration of vitamin D has been associated with obesity [13,14], the independent role of vitamin D in PCOS has been challenging to assess [15]. 

Elevated androgen levels in PCOS cause unfavorable derangements in adipose tissue and in glucose metabolism [16]. It has been proposed that the connection between vitamin D and PCOS arises from the endocrine pathways affected in PCOS, such as sex hormone synthesis and insulin secretion [17,18]. However, clinical trials involving vitamin D supplementation in women with PCOS have shown conflicting or weak results in terms of improving insulin sensitivity and other metabolic factors, such as low-grade inflammation and androgen levels [19,20,21,22].

Previously, studies of vitamin D levels in PCOS have mostly included small clinical samples, while studies on large, unselected populations have been lacking. The interpretation of studies is complicated because only a few have assessed the possible presence of the important confounders of vitamin D status, for example, body mass index (BMI), lifestyle, seasonal, and latitudinal effect.

The objective of this prospective population-based study, therefore, was to evaluate vitamin D status in 31-year-old women with self-reported PCOS symptoms and/or diagnosed PCOS, compared with non-PCOS controls. A comprehensive range of potential confounding factors of vitamin D and PCOS were assessed to test the hypothesis that women with self-reported PCOS are more likely to be vitamin D insufficient than are controls, and that vitamin D levels are negatively associated with BMI in PCOS.

## 2. Materials and Methods 

### 2.1. Study Population

**Figure 1:**
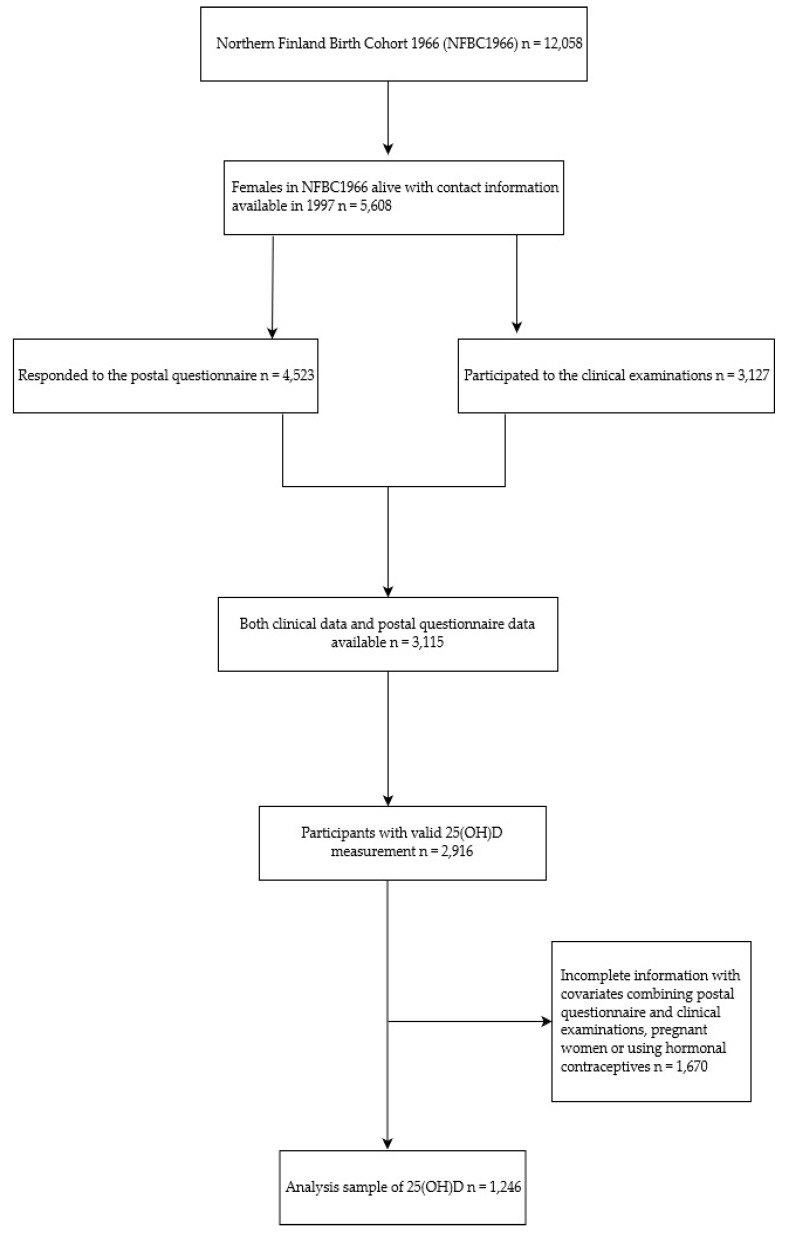
Flowchart showing the measured 25-hydroxyvitamin D (25(OH)D) samples in the female study population of the Northern Finland Birth Cohort 1966 (NFBC1966).

The study population was drawn from the Northern Finland Birth Cohort 1966 (NFBC1966), which includes all live births that occurred in 1966 in northern Finland (*n* = 12,058; 5889 females; 96.3% of all births in Oulu and Lapland) [23]. Initial data collection occurred at the 24th week of gestation, and participants were then examined at birth, with further examinations at the ages of 1, 14, 31, and 46. This study utilized the data from the two latter examinations. A postal questionnaire (collecting information about social background, behavior, work status, medical history, and previously diagnosed diseases) was sent to participants at the age of 31. The questionnaire was sent to 5608 women, and 4523 (81%) responded (Figure 1). Alongside the questionnaire, participants with an address in northern Finland and the Helsinki metropolitan area were invited to attend a clinical examination. In total, 3127 (77% of the target population) attended a clinical examination, which included several different anthropometric and laboratory measurements. Postal questionnaire information and clinical data were available from a total of 3115 women. At the age of 46, 5123 women received and 3732 (73%) answered an updated postal questionnaire, which also included questions about medical history and previously diagnosed diseases. Permission was obtained from the ethical committee of the Northern Ostrobothnia hospital district (94/2011, 12/2003), and the Helsinki declaration and national guidelines were followed. A written informed consent was obtained from all participants.

### 2.2. Clinical Assessments

For the questionnaire conducted with 31-year-old participants, the existence (or non-existence) of PCOS-related symptoms was ascertained with the following questions: ‘Is your menstrual cycle often (more than twice a year) over 35 days?’ and ‘Do you have excessive body hair?’ Of those women who answered the questionnaire at the age of 31, 125 (4.2%) women answered ‘Yes’ to both the oligo-amenorrhea and hirsutism questions. The two questions had been previously validated to accurately identify PCOS cases as they present the typical hormonal and metabolic features of the syndrome [24,25,26,27]. At the age of 46, 181 (5.0%) women answered ‘Yes’ to the question, ‘Have you ever been diagnosed as having polycystic ovaries (PCO) and/or polycystic ovary syndrome (PCOS)?’ Overall, the self-reported PCOS population was formed from the combination of affirmative answers to the above questions from these two age groups (*n* = 280). The control population included all other women without PCOS symptoms at the age of 31 and who also answered ‘No’ to the PCOS question at the age of 46 (*n* = 1573). Because pregnancy and the use of hormonal contraceptives might cause oligo-amenorrhea and improve irregular cycles, those participants were excluded from the data at 31 years (*n* = 1488), along with participants who did not give permission to use their data (31 years, *n* = 44; 46 years, *n* = 20) [27].

### 2.3. Laboratory Measurements

In 1997, when participants were 31 years old, blood samples were drawn after overnight fasting between 08:00 and 11:00 a.m. The samples were stored at −70 °C before being analyzed. Serum samples were defrosted and measured in four batches between the years 2008 and 2009. Serum 25-hydroxyvitamin D levels (25(OH)D), the major reflector of vitamin D reserves, were measured with a reliable and commonly used method, high-performance liquid chromatography–tandem mass spectrometry (LC–MS/MS; Elstree, Hertfordshire, UK), and were validated with Diasorin (Stillwater, MN, USA) radioimmunoassay (RIA) (which has a ^125^I-labeled tracer) [28,29]. The total serum 25(OH)D concentration was defined by the sum of 25(OH)D2 and 25(OH)D3. For the 25(OH)D analyses, measured with LC–MS/MS, the Vitamin D External Quality Assessment Scheme (DEQAS, Elstree, Hertfordshire, UK) was used to validate the quality and accuracy of the method. The coefficient of variation (CV) was less than 16% across the working range of the assay. The measurement techniques and methods have been described previously in detail [28,29,30]. The cut-off levels for 25(OH)D were defined based on Institute of Medicine (IOM) guidelines as ≤30 nmol/L (deficiency), 30–50 nmol/L (insufficiency), and ≥50 nmol/L (sufficient level) [31,32,33]. A total of 2916 women had valid 25(OH)D measurements and, after the exclusions described above, 1246 women (self-reported PCOS, *n* = 194; controls, *n* = 1052) remained. Serum concentrations of testosterone, high sensitive C-reactive protein (hs-CRP), fasting serum insulin, and plasma glucose were all assayed as previously described in NordLab Oulu (former name, Oulu University Hospital Laboratory, Oulu, Finland), a testing laboratory (T113) accredited by the Finnish Accreditation Service (FINAS) (EN ISO 15189) [24,27]. To estimate insulin resistance, homeostatic model assessment of insulin resistance (HOMA-IR) values were calculated from fasting plasma glucose (fP-Gluc) and serum insulin (fS-Ins) levels ([fP-Gluc * fS-Ins]/22.5) [34]. BMI was calculated using clinical examination data at the age of 31. The data from the postal questionnaires was used if clinical weight and height measurements were missing [27]. Self-reported and clinically measured BMI have been verified to give the same results [27].

### 2.4. Covariates

To assess the impact of solar vitamin D, the calendar year was divided into high (summer (1 June–30 August) and autumn (1 September–31 October)) and low (winter (1 November–31 March) and spring (1 April–31 May)) vitamin D seasons, based on the time of clinical assessment of each woman [30,35]. Information about the participants’ place of residence in 1997, taken from the population register center, was used to categorize those living at the latitude of 60° N (Helsinki and surrounding areas), 65° N (the city of Oulu) and >65° N (elsewhere in the northernmost provinces of Oulu and Lapland) [30].

The postal questionnaire information was used to clarify confounding lifestyle factors [30]. The consumption of various food items in the previous six months was used to calculate diet scores on a scale of 0–5: scores denoted either a healthy diet (≤3 points) or an unhealthy diet (4–5 points). The food questions included 32 products classified as grain and milk products, meat, vegetables, fruits, and others (e.g., convenience food, sweets) [36]. Alcohol consumption was categorized as follows: abstainer, low-risk drinker (≤20 g/day), and at-risk drinker (>20 g/day) [36,37]. Smoking was classified as follows: non-smoker, former/occasional smoker, and active smoker. Physical activity was calculated as the metabolic equivalent of task (MET) scores in hours per week from the frequency and duration of leisure time activities (3 METs = light physical activity, 5 METs = brisk physical activity) [38].

Socioeconomic status (SES) was classified into five categories based on occupation (professional, skilled worker, unskilled worker, farmer, and others). History of infertility treatment was defined based on the questionnaire items, given at the age of 31, ‘Have you ever been examined for infertility?’ and ‘Have you been treated for infertility?’.

### 2.5. Statistics

Outliers were defined as observations that lie at an abnormal distance from other values [39]. To find the extreme outliers of 25(OH)D measurements, the interquartile range (IQR) was calculated. If the 25(OH)D level was above the 75th percentile + 1.5 * IQR, the 25(OH)D measurement was excluded [39]. After exclusion, 192 of the 25(OH)D measurements in the self-reported PCOS group and 1048 in the control group remained. The independent samples t-test or nonparametric Mann–Whitney U test was used to compare continuous variables. Based on the distribution of total 25(OH)D, the quartile cut-off points were 39.0, 49.5, and 59.7 nmol/L. Pearson’s χ2 test was used for comparing the distributions of categorical variables (i.e., 25(OH)D quartiles, latitude, season of blood sampling, diet score, alcohol consumption, smoking habit, SES, and infertility treatment) across the self-reported PCOS and control groups. The general linear regression model was used to define mutually adjusted associations of different exposures with 25(OH)D levels. The variables of the model were selected on the grounds of statistically significant results from the abovementioned tests and the factors that are known to be associated with 25(OH)D level [30]. The variables in the final model were self-reported PCOS, BMI, season of blood sampling, latitude, and vitamin D batch effect. *p*-values of <0.05 were considered statistically significant. The statistical analyses were executed using IBM SPSS Statistics for Windows, Version 25 (IBM Corp., Armonk, NY, USA). The forest plot was executed with Rstudio (version 1.0.143, https://www.rstudio.org).

## 3. Results

The characteristics of the study population are shown in Table 1. The mean 25(OH)D level did not differ between women with self-reported PCOS (50.35 ± 13.51 nmol/L) and the non-symptomatic controls (48.30 ± 13.37 nmol/L, *p* = 0.051, Table 2).

In the PCOS group, 25(OH)D levels were deficient in 12 women (6.3%) and insufficient in 87 women (45.3%). In the control group, these levels were deficient in 93 women (8.9%) and insufficient in 473 women (45.1%, *p* = 0.465). In women with self-reported PCOS, 38 (19.8%) had 25(OH)D levels in the lowest quartile (<39.0 nmol/L) and 57 (29.7%) had levels in the second lowest quartile (39.1–49.5 nmol/L). Within the control group, 299 (28.5%) had 25(OH)D levels in the lowest quartile and 251 (24.0%) had levels in the second lowest quartile (*p* = 0.058, Table 2). Women with self-reported PCOS were more likely to live in northern latitudes in comparison to controls (*p* = 0.002). Other background characteristics did not differ between the two groups (i.e., season of blood sampling, diet score, alcohol consumption, smoking habits, physical activity, or SES).

Women with self-reported PCOS had a higher mean body mass index (BMI) (26.23 ± 6.05 vs. 23.61 ± 4.19 kg/m^2^, *p* < 0.001, Table 3), testosterone (1.40 ± 0.65 vs. 1.03 ± 0.43 nmol/L, *p* < 0.001), and HOMA-IR (1.23 ± 0.77 vs. 1.00 ± 0.43, *p* < 0.001) concentrations than in the controls. High-sensitivity C-reactive protein (hs-CRP) levels were almost twice as high in women with self-reported PCOS than in the controls (2.62 vs. 1.63 mg/L, *p* < 0.001). Women with self-reported PCOS were also treated more often for infertility (23.5% vs. 5.5%, *p* < 0.001). A sub-analysis with the self-reported PCOS participants showed no significant difference in testosterone concentrations between the vitamin D quartiles. For women with PCOS in the lowest 25(OH)D quartile (*n* = 37) testosterone was 1.20 nmol/L, in the second quartile (*n* = 56) 1.40 nmol/L, in the third quartile (*n* = 51) 1.44 nmol/L, and in the highest 25(OH)D quartile (*n* = 42) 1.48 nmol/L, respectively (*p* < 0.239).

### Association of 25(OH)D Level and Self-Reported PCOS

**Figure 2:**
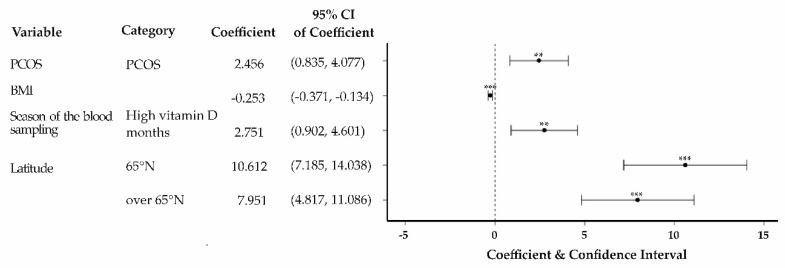
Associations of 25-hydroxyvitamin D (25(OH)D) levels and PCOS with relevant exposures in linear regression model. The model was adjusted with BMI, season of the blood sampling, latitude, and vitamin D laboratory batch effect. The reference classes for categorized variables were as follows: PCOS = controls; BMI as a continuous variable; season of the blood sampling = low vitamin D months; latitude = 60° N. *** *p* < 0.001, ** *p* < 0.01. 60° N = Helsinki and surrounding areas, 65° N = the city of Oulu, and >65° N = the northernmost provinces of Oulu and Lapland; PCOS = polycystic ovary syndrome; BMI = body mass index; N = north; CI = confidence interval.

The mutually adjusted multivariable linear regression model (Figure 2) shows that 25(OH)D level was positively associated with self-reported PCOS (β = 2.46, 95% confidence interval (CI) 0.84 to 4.08, *p* = 0.003, r^2^ = 0.45) when compared with controls. However, BMI was negatively associated with 25(OH)D levels in the same model (β = −0.25, 95% CI −0.37 to −0.13, *p* < 0.001). The 25(OH)D levels were the highest in women living at the latitude of 65°N (β = 10.61, 95% CI = 7.19 to 14.04, *p* < 0.001) and lowest in those living at 60° N. The 25(OH)D levels were higher in high-sunlight months (i.e., June, July, August, September, and October) compared to low-sunlight months (β = 2.75, 95% CI = 0.90 to 4.60, *p* = 0.004). Adjustment for testosterone, HOMA-IR, and hs-CRP did not change the results: self-reported PCOS still had a positive association with 25(OH)D (β = 2.39, 95% CI 0.65 to 4.13, *p* = 0.007, r^2^ = 0.45).

## 4. Discussion

Our large general population dataset evaluated serum 25(OH)D concentrations in women with self-reported PCOS versus those in non-symptomatic controls. The 25(OH)D levels were positively associated with self-reported PCOS, after adjusting for multiple confounding factors. Still, a considerable number of women in both groups had insufficient 25(OH)D levels [31]. Despite this, the mean 25(OH)D concentration in the self-reported PCOS participants was above the normal range.

Given that hypovitaminosis D is connected to the onset of chronic diseases and that the deficiency may interfere with the normal physiology of the human body [40], we anticipated the women with PCOS in our population-based cohort would have lower vitamin D levels compared with the non-PCOS controls. Women with PCOS did show the accumulation of several metabolic risk factors, namely, high BMI, HOMA-IR, and hs-CRP. Moreover, studies from this cohort report a higher rate of type 2 diabetes (T2D), hypertension, and dyslipidemia in affected women [27,41,42]. Our results revealed that, despite these metabolic derangements, women with PCOS have an adequate vitamin D status compared with the controls.

Prior studies have shown PCOS to be a risk factor for vitamin D insufficiency, high BMI, and metabolic syndrome [3,6,43], which coincide with metabolic disturbances in PCOS [15,44]. Women with PCOS with a high BMI have more insufficient 25(OH)D levels compared with their normal-BMI counterparts [6,15,44]. Considering the higher BMI and metabolic derangements in our PCOS population, we found that the mean 25(OH)D levels in the women with PCOS were still in a normal range, although they were almost at the cut-off level of 25(OH)D insufficiency. A considerable number of women were vitamin D insufficient, but a similar finding was observed in the non-PCOS controls, in line with previous observations [9,45].

Previous studies have shown inconsistent results in terms of vitamin D levels in PCOS women, which have mainly been in opposition with the present results [16,44,46], although this has not been the case in all studies [9,45]. Studies reporting lower vitamin D levels in women with PCOS have been conducted with fairly small sample sizes, recruiting cases from infertility clinics without considering substantive confounding factors [9,16,46]. Recent studies suggest that lower 25(OH)D levels are associated with prolonged menstrual cycles in healthy women [47] and that vitamin D supplementation promotes follicle development and restores menstrual cycles in women with PCOS [6,48]. Women with infertility may be a particularly relevant population to study regarding vitamin D deficiency. However, some of the prior studies have suggested, that excess levels of 25(OH)D may also have adverse effects to the fertility [49]. Thus, vitamin D supplementation should be used with evidence-based and appropriate dosing [49].

One of the largest studies, including 639 women with PCOS and 449 controls, reported lower vitamin D levels in anovulatory women with PCOS compared with healthy controls [44]. In that study, PCOS cases presented with anovulatory infertility, and they were recruited from outpatient clinics, in contrast to our population-based cohort. It is possible that the PCOS phenotype was more severe in the study by Krul-Poel et al. than in our study because the subject data were obtained from an infertility clinic rather than from a general population based on self-reported PCOS, even though both studies included women who met the Rotterdam criteria [50]; in addition, in both studies, the PCOS group had a higher BMI than did the control group. In contrast to our control population, the controls in that study were fertile and had undergone a normal delivery less than 1.5 years ago; they may have had a better health status than did our population-based controls. As the samples were obtained close to pregnancy and those who were likely nursing, the use of vitamin D supplementation is more likely in this population and may explain the discrepancies. 

Studies have demonstrated that vitamin D might have anti-inflammatory effects and beneficial contributions to glucose metabolism [9,22,51,52,53]. However, higher 25(OH)D levels in the PCOS group did not result in lower hs-CRP levels. Whether this contributes to a lower prevalence of cardiovascular disease (CVD) in women with PCOS than is expected from their risk profile remains to be investigated [54]. The health-promoting effects in this patient group may only be achieved by higher vitamin D levels, and women with PCOS still need education to ensure adequate vitamin D supplementation. 

Hyperandrogenism and insulin resistance are the substantial factors causing the metabolic disturbances in PCOS [17,55]. Androgen levels are noted to be reduced in women with PCOS, when influencing the insulin metabolism with medication such as insulin sensitizer [55]. Vitamin D might also have beneficial effects to the insulin responsiveness and androgen levels in PCOS [6,17,18,56]. Nonetheless, a recent study indicated, vitamin D supplementation may not protect from T2D in prediabetic patients [57], explaining why vitamin D did not inhibit the elevation of HOMA-IR in our PCOS population. Our women with PCOS had higher testosterone concentrations than the controls. The sub-analysis did not however show significant difference in testosterone levels between the vitamin D quartiles as would be expected based on previous observations [6,56].

The season of blood sampling is associated with vitamin D levels [58,59]. Despite a higher proportion of self-reported PCOS women living in the northernmost latitudes of Finland, increasing the risk for vitamin D insufficiency, the mean 25(OH)D levels were sufficient compared to the non-PCOS controls. The background characteristics associated with vitamin D insufficiency did not differ between the two groups and do not explain the result in the present study. Nutrition habits contributing to weight gain [60] were not different between our study groups.

The bioavailability of vitamin D might decrease when sequestered to adipose tissue, explaining the negative association between BMI and vitamin D levels, and the differences between the two groups [15]. Further, the actions of vitamin D are mediated through VDRs [18], which modulate the expression of several genes related to glucose and lipid metabolism [44]. PCOS may be considered as a multigenic disease with a close relation to the metabolic disturbances [17]. VDR polymorphism has been associated with elevated risk for PCOS, possibly through affecting insulin and testosterone levels [18,61,62,63,64]. Our findings may indicate 25(OH)D resistance in women with PCOS, which could be due to vitamin D gene polymorphism [18,65]. More studies are warranted to explore the genetic associations between PCOS and vitamin D. 

A key strength of our study is that it is the largest study to date assessing vitamin D levels in women with PCOS using a population-based setting. The participation rate in the cohort is high and the participants have the same ethnic and genetic background. In addition, the narrow age range of the present population controlled the factor that vitamin D levels are affected by age [10]. Due to the original cohort study setting and wide range of data, it was possible to include several potential confounding factors for vitamin D and PCOS in the analyses. Previous studies used selected populations from hospitals or private clinics, introducing a selection bias. The limitations of our study include the use of self-reported symptoms and diagnosis of PCOS. However, other studies using the present cohort have validated that self-reported symptoms and PCOS diagnosis are reliable in successfully identifying women with PCOS [24,25,26,27,42]. Because hormonal contraceptives can be used to treat PCOS symptoms and those using such regiments were excluded from the study, the true number of PCOS participants may have been underestimated [66]. However, women with higher estrogen levels and those using combined hormonal contraceptives tend to have higher 25(OH)D levels [30,67]. If women with PCOS using hormonal contraceptives were to be included in our study, the results would likely be strengthened [30,58]. Only 10.2% of the total Finnish population used vitamin D supplementation in 2000 [68]. Therefore, as the use of supplementation was uncommon at the time of the blood sampling, the lack of information on vitamin D supplementation is an acceptable limitation. Furthermore, the nationwide vitamin D fortification of dairy products and margarine occurred in Finland after the blood sampling from 2002 onward [69].

## 5. Conclusions

In our cohort, women with PCOS showed no greater propensity to vitamin D insufficiency than the controls. Our model with various potential confounding factors suggested that mean vitamin D levels were higher in the PCOS group than those in the control group. Based on our findings, vitamin D levels had no correlation with substantial positive health effects in the alleviation of metabolic syndrome or CVD-related risk factors. However, we recommend ensuring sufficient vitamin D levels in women with PCOS and especially in overweight and obese individuals.

## Figures and Tables

**Table 1:** Background characteristics of the study participants: women with self-reported polycystic ovary syndrome (PCOS) and non-PCOS controls.

Characteristic	PCOS (*n* = 280)	Controls (*n* = 1573)	*p*-Value
Daylight			
Season of the blood sampling, n (%) ^a^			0.554 ^e^
High vitamin D months	136 (66.7)	720 (64.5)	
Low vitamin D months	68 (33.3)	396 (35.5)	
Latitude, n (%) ^b^			0.002 ^e^
65° N	43 (17.5)	268 (17.0)	
>65° N	147 (59.7)	775 (49.3)	
60° N	56 (22.8)	530 (33.7)	
Lifestyle			
Diet score, n (%) ^c^			0.869 ^e^
0–1	122 (44.8)	689 (43.8)	
2–3	130 (47.8)	777 (49.4)	
4–5	20 (7.4)	107 (6.8)	
Alcohol consumption, n (%)			0.199 ^e^
Abstainer	35 (13.1)	182 (11.9)	
Low-risk drinker (≤20 g/day)	213 (79.8)	1275 (83.4)	
At-risk drinker (≥20 g/day)	19 (7.1)	72 (4.7)	
Smoking, n (%)			0.262 ^e^
Active smoker	72 (26.7)	352 (22.4)	
Former/Occasional smoker	63 (23.3)	414 (26.4)	
Non-smoker	135 (50.0)	803 (51.2)	
Physical activity, (mean ± SD) ^d^	14.31 ± 12.36	15.48 ± 13.59	0.182 ^f^
Socioeconomic status, n (%)			0.114 ^e^
Professional	50 (18.4)	365 (23.3)	
Skilled worker	114 (41.9)	674 (43.0)	
Unskilled worker	44 (16.2)	218 (13.9)	
Farmer	11 (4.0)	33 (2.1)	
Others	53 (19.5)	277 (17.7)	

^a^ High vitamin D months: summer (1 June–30 August) and autumn (1 September–31 October). Low vitamin D months: winter (1 November–31 March) and spring (1 April–31 May). ^b^ Latitudes: 60° N = Helsinki and surrounding areas, 65° N = the city of Oulu, and >65° N = the northernmost provinces of Oulu and Lapland. ^c^ Diet scores: a healthy diet (≤3 points) and an unhealthy diet (4–5 points). Calculated from the consumption of different food items. ^d^ The metabolic equivalent of task of physical activity (MET) scores in hours per week (frequency and duration of leisure time activities). ^e^ Pearson’s chi-squared test. ^f^ Fisher’s exact test.

**Table 2:** Serum 25-hydroxyvitamin D (25(OH)D) levels and distributions in women with polycystic ovary syndrome (PCOS) and non-PCOS controls.

25(OH)D Status	PCOS (*n* = 192)	Controls (*n* = 1048)	*p*-Value
25(OH)D nmol/L (mean ± SD)	50.35 ± 13.51	48.30 ± 13.37	0.051
25(OH)D levels			0.465
≤30.0 nmol/L, n (%)	12 (6.3)	93 (8.9)	
30.0–50.0 nmol/L, n (%)	87 (45.3)	473 (45.1)	
≥50.0 nmol/L, n (%)	93 (48.4)	482 (46.0)	
25(OH)D quartiles			0.058
<39.0 nmol/L, n (%)	38 (19.8)	299 (28.5)	
39.0–49.5 nmol/L, n (%)	57 (29.7)	251 (24.0)	
49.5–59.7 nmol/L, n (%)	51 (26.6)	280 (26.7)	
>59.7 nmol/L, n (%)	46 (23.9)	218 (20.8)	

**Table 3:** Clinical and biochemical features in women with polycystic ovary syndrome (PCOS) and non-PCOS controls.

Feature	PCOS (*n* = 193–268)	Controls (n =1070–1560)	*p-*Value
BMI, kg/m^2 a^	26.23 ± 6.05	23.61 ± 4.19	<0.001
Testosterone, nmol/L (mean ± SD)	1.40 ± 0.65	1.03 ± 0.43	<0.001
HOMA-IR, (mean ± SD) ^b^	1.23 ± 0.77	1.00 ± 0.43	<0.001
hs-CRP, mg/L (mean ± SD)	2.62 ± 4.01	1.63 ± 3.41	<0.001
Infertility treatment, n (%) ^c^			<0.001
Yes	63 (23.5)	83 (5.5)	
No	205 (76.5)	1437 (94.5)	

^a^ BMI calculated as weight (kg)/(height (m))^2^. ^b^ HOMA-IR calculated as (fP-Gluk * fS-Ins)/22.5. ^c^ Calculated from questions “Have you ever been examined for infertility?” and “Have you been treated for infertility?”. HOMA-IR = homeostatic model assessment of insulin resistance; hs-CRP = high-sensitivity c-reactive protein; fP-Gluk = fasting plasma glucose; fS-Ins = fasting serum insulin.
